# Maturation of Calcium-Dependent GABA, Glycine, and Glutamate Release in the Glycinergic MNTB-LSO Pathway

**DOI:** 10.1371/journal.pone.0075688

**Published:** 2013-09-19

**Authors:** Javier Alamilla, Deda C. Gillespie

**Affiliations:** Department of Psychology, Neuroscience & Behaviour, McMaster University, Hamilton, Ontario, Canada; Dalhousie University, Canada

## Abstract

The medial nucleus of the trapezoid body (MNTB) is a key nucleus in high-fidelity temporal processing that underlies sound localization in the auditory brainstem. While the glycinergic principal cells of the MNTB project to all primary nuclei of the superior olive, during development the projection from MNTB to the lateral superior olive (LSO) is of interest because this immature inhibitory projection is known to undergo tonotopic refinement during an early postnatal period, and because during this period individual MNTB terminals in the LSO transiently release glycine GABA and glutamate. Developmental changes in calcium-dependent release are understood to be required to allow various auditory nuclei to follow high frequency activity; however, little is known about maturation of calcium-dependent release in the MNTB-LSO pathway, which has been presumed to have less stringent requirements for high-fidelity temporal following. In acute brainstem slices of rats age postnatal day 1 to 15 we recorded whole-cell responses in LSO principal neurons to electrical stimulation in the MNTB in order to measure sensitivity to external calcium, the contribution of different voltage-gated calcium channel subtypes to vesicular release, and the maturation of these measures for both GABA/glycine and glutamate transmission. Our results establish that release of glutamate at MNTB-LSO synapses is calcium-dependent. Whereas no significant developmental changes were evident for glutamate release, GABA/glycine release underwent substantial changes over the first two postnatal weeks: soon after birth L-type, N-type, and P/Q-type voltage-gated calcium channels (VGCCs) together mediated release, but after hearing onset P/Q-type VGCCs predominated. Blockade of P/Q-type VGCCs reduced the estimated quantal number for GABA/gly and glutamate transmission at P5–8 and the frequency of evoked miniature glycinergic events at P12–15, without apparent effects on spontaneous release of neurotransmitter, supporting a model in which P/Q-type VGCCs are required for mature synchronous synaptic transmission, but not for spontaneous vesicle release.

## Introduction

The lateral superior olive (LSO) nucleus in auditory brainstem computes interaural intensity differences necessary for localizing sound [1], [2]. The principal cells of the LSO are large fusiform projection neurons that receive glutamatergic inputs from the ipsilateral anteroventral cochlear nucleus (AVCN) [3], [4], and glycinergic inputs from the medial nucleus of the trapezoid body (MNTB), which is itself driven by inputs from the contralateral ear. Postnatal tonotopic refinement within the LSO makes this nucleus an attractive model system for studies of how inhibitory circuits are sculpted through developmental refinement.

The major postnatal period of circuit refinement in the MNTB-LSO pathway – as measured by changes in functional synapse number and strength – occurs before hearing onset, or before about postnatal day 12 (P12) in rats [5]. Before hearing onset, as is common at other immature glycinergic synapses, MNTB terminals also release GABA [6], [7], and the GABA and glycine released by MNTB terminals exert depolarizing effects on postsynaptic LSO neurons [8], [9]. Interestingly, during this period MTNB terminals transiently express both the vesicular glutamate transporter 3 (VGLUT3) and the calcium sensor Synaptotagmin 1, and stimulation within the MNTB results in release of not only GABA and glycine but also glutamate in the LSO [10], [11].

Circuit maturation in general also depends on modifications in synaptic machinery, importantly on maturation of Ca^++^-dependent release due to changes in coupling to specific types of voltage-gated Ca^++^ channels (VGCCs) [12], which in turn can strongly influence short-term synaptic plasticity [13]. Short-term plasticity not only tunes information transfer at mature synapses [14] affecting circuit-level processing [15], but also during the period of developmental refinement could exert longer-term effects by filtering the patterns of activity thought to be directly responsible for shaping the nascent circuit (for review, see [16]).

The large, diverse family of VGCCs includes several subfamilies. Neurotransmission at mature central synapses is mediated primarily by the Ca_v_2 family, which includes the P/Q-type (Ca_v_2.1), which is sensitive to ω-agatoxin IVA [17], [18], the N-type (Ca_v_2.2), which is sensitive to ω-conotoxin GVIA [19], and the R-type (Ca_v_2.3), which is resistant to most subtype-specific Ca^++^ channel blockers [20]. The L-type (Ca_v_1) family, which is sensitive to dihydropyridines [21], is found most often at soma and dendrites, while the low-voltage-activated, peptide-resistant T-type (Ca_v_3 family) [22] contributes to burst formation and pacemaking. A developmental maturation of specific VGCCs, generally trending toward expression of P/Q-type, has been found in several areas [23]–[26].

It has been proposed that the phase-processing circuit of MNTB and medial superior olive (MSO) may require the highest temporal fidelity and most reliable synaptic transmission in the nervous system, together with specific VGCCs to support that level of transmission [24], whereas intensity-processing circuits of the LSO may be able to dispense with the ability to follow rapid spike trains with high fidelity. At excitatory terminals in the MNTB and at inhibitory terminals in the MSO, mature VGCC expression appears to consist of nearly pure P/Q and mostly P/Q, respectively [27], [28]. Recordings in the MNTB-LSO pathway in mouse, by contrast, suggest that after hearing onset, P/Q channels predominate only slightly over L- and N-type [29]. However, despite several studies of postsynaptic VGCC development [30], [31], virtually nothing is known about development of presynaptic calcium channels in the MNTB-LSO pathway before hearing onset, and so to better understand the processes involved in maturation of release, we examined effects of calcium concentration and functional expression of specific VGCCs on GABA/gly transmission, as well as maturation (if any) of glutamate release at MNTB terminals in postnatal rat.

## Materials and Methods

Tissue from Sprague-Dawley rats born to animals bred on site or shipped pregnant (Charles River Laboratories, Wilmington, MA) was used throughout.

### Ethics Statement

All procedures were performed in accord with Canadian Council on Animal Care guidelines and were previously approved by the Animal Review Ethics Board of McMaster University (AUP 12-05-16).

### Slice preparation

Rat pups at ages postnatal day 1–15 (P1–15) were deeply anesthetized (isoflurane), and the brain was quickly removed and placed in ice-cold artificial cerebrospinal fluid (ACSF) containing (in mM): NaCl, 124; MgSO_4_, 1; KCl, 5; KH_2_PO_4_, 1.25; dextrose, 10; NaHCO_3_, 26; CaCl_2_, 2; kynurenic acid, 1, pH 7.2, 300±5 mOsm, oxygenated with 95% O_2_/5% CO_2_. Coronal sections (300 µm) were obtained at the vibratome and slices containing the MNTB-LSO pathway were allowed to recover at room temperature (19–21°C) for 1 h in a humidified, oxygenated, interface chamber with fresh ACSF. The slice was then placed in the recording chamber and continuously perfused in oxygenated, kynurenic acid-free, ACSF (5 ml/min). Using IR-DIC, principal cells of the LSO were visually identified by their morphology and position within the LSO.

### Electrophysiology

Whole-cell recordings (Axoclamp 700B, Axon Instruments, Sunnyvale, CA) were made at elevated temperature (32–34°C in bath, maintained with ThermoClamp inline heater, AutoMate Scientific, Berkeley, CA) using borosilicate electrodes of resistance 1.5–2.5 MΩ (P97, Sutter Instruments). Series resistance was compensated (80%) and the seal monitored throughout each experiment. Cells were discarded if input resistance was <150 MΩ or if access resistance exceeded 25 MΩ or changed by >15%. Recordings were sampled at 10 kHz and filtered at 5 kHz (pCLAMP, Digidata 1400, Axon Instruments). Stimulating electrodes (1 MΩ patch electrodes containing ACSF) were placed in the MNTB. Stimulus strength was adjusted to the lowest intensity that produced a reliable response (Master 8 with Iso-Flex SIU, Jerusalem, Israel).

### General release properties

The glutamate component was isolated by bath application of picrotoxin (50 µM, Tocris) and strychnine (10 µM, Sigma). Cyclothiazide (100 µM, Ascent) was used in all recordings involving glutamate transmission in order to minimize AMPA receptor desensitization. The GABA/gly component was isolated by application of CNQX (5 µM, Tocris). The internal solution used to study release properties contained (in mM): D-gluconic acid, 64; CsOH, 64; EGTA, 11; CsCl, 56; MgCl_2_, 1; CaCl_2_, 1; HEPES, 10; GTP-Na, 0.3; ATP-Mg, 4 and QX-314, 5; Cl_Eq_∼−25 mV (32°C). Normal Ca^++^ in the perfusate was defined as 2.0 mM (ACSF given above); adjustments to Ca^++^ concentration were matched by compensatory adjustments to external Na^+^ concentration to minimize changes in osmolarity.

### Contribution of specific VGCC subunits

Voltage-gated Ca^++^ channels were blocked pharmacologically, using the Ca_v_1.1–Ca_v_1.4 (L-type) antagonist nitrendipine (10 µM, Tocris), the Ca_v_2.1 (P/Q-type) antagonist ω-agatoxin IVA (200 µM, Alomone), and the Ca_v_2.2 (N-type) antagonist ω-conotoxin GVIA (1 µM, Alomone), applied in random order. As we lack a selective antagonist for Ca_v_2.3 (R-type) channels, the general VGCC antagonist Cd^++^ (200 µM) was applied after the three subtype-selective antagonists. Bumetanide (10 µM, Tocris) was added to minimize dynamic regulation of chloride co-transporters in whole cell records [32]. To compare the contributions of different VGCC subunits to GABA/gly and glutamate transmission within the same cell, the intracellular solution contained (in mM): D-gluconic acid, 118; CsOH, 118; EGTA, 11; CsCl, 2; MgCl_2_, 1; CaCl_2_, 1; HEPES, 10; GTP-Na, 0.3, ATP-Mg, 4 and QX-314, 5; Cl_Eq_∼−90 mV (32°C). Single stimuli were delivered to the MNTB at 0.1 Hz. First, baseline responses (10 traces) were acquired for each component, recording GABA/gly responses at the reversal potential for the AMPA current (0 mV) and glutamate responses at the reversal potential for Cl- (−90 mV). The three selective blockers were then applied sequentially and cumulatively, in randomly-assigned order, with each blocker present for at least 6 minutes, depending on order of application. The glutamatergic current was monitored for 6 minutes after administration of each blocker (L-, N- and P/Q-type), and the last 10 traces were acquired to assess the drug effect. The holding potential was switched to 0 mV just long enough to record 10 GABA/gly responses, then was returned to −90 mV and the next blocker was applied. The proportional contribution of each VGCC subtype to neurotransmitter release was inferred from the amount by which each specific blocker decremented the previous response. In experiments utilizing VGCC toxins, cytochrome-c is typically added to saturate non-specific binding sites [33]. We compared a subset of experiments conducted without cytochrome-c and found that whereas the presence of cytochrome-c (0.1 mg/ml, Sigma) reduced response variability slightly, it produced no significant differences in mean response (Mann-Whitney U test, data not shown). We have therefore included data from experiments conducted both in the presence and absence of cytochrome-c.

### Estimate of quantal number

Cumulative amplitude of the responses elicited by a 20-pulse 100 Hz stimulus train was plotted against stimulus number [34]. The y-intercept of the linear regression to the last 8 points of this function was used to estimate the size of the readily releasable pool, and this value divided by the average amplitude of the spontaneous PSC (sPSC) provided an estimate of quantal number.

### Analysis

Offline analysis was performed using Clampfit (Molecular Devices, Silicon Valley, CA), Origin (OriginLab, Northampton, MA), MiniAnalysis (Synaptosoft, Decatur, GA), and custom-written Matlab (MathWorks, Natick, MA) programs. Data were tested for normality (Shapiro-Wilk) and for homogeneity of variance (Levene), and parametric or non-parametric (Wilcoxon, Mann-Whitney, Kruskal-Wallis and two-way ANOVA) statistical tests to assess significance at alpha levels of 0.05, 0.005 and 0.0005 were performed using Prism (GraphPad, San Diego, CA) and SPSS (IBM Corporation, Armonk, NY). Population statistics are reported as mean±SEM.

## Results

We used whole-cell patch recordings in acute slice to examine maturation of release for GABA/gly, as well as for glutamate, from MNTB terminals within the LSO. All recordings were made in voltage-clamp mode from principal cells of the LSO, in response to electrical stimulation in the MNTB. We isolated the GABA/gly component by recording in the presence of CNQX (5 µM), and the glutamate (AMPA) component by recording in the presence of picrotoxin and strychnine (50 µM and 10 µM). Cyclothiazide (100 µM) was included in all recordings of AMPA-mediated currents to prevent AMPA-receptor desensitization.

**Figure 1:**
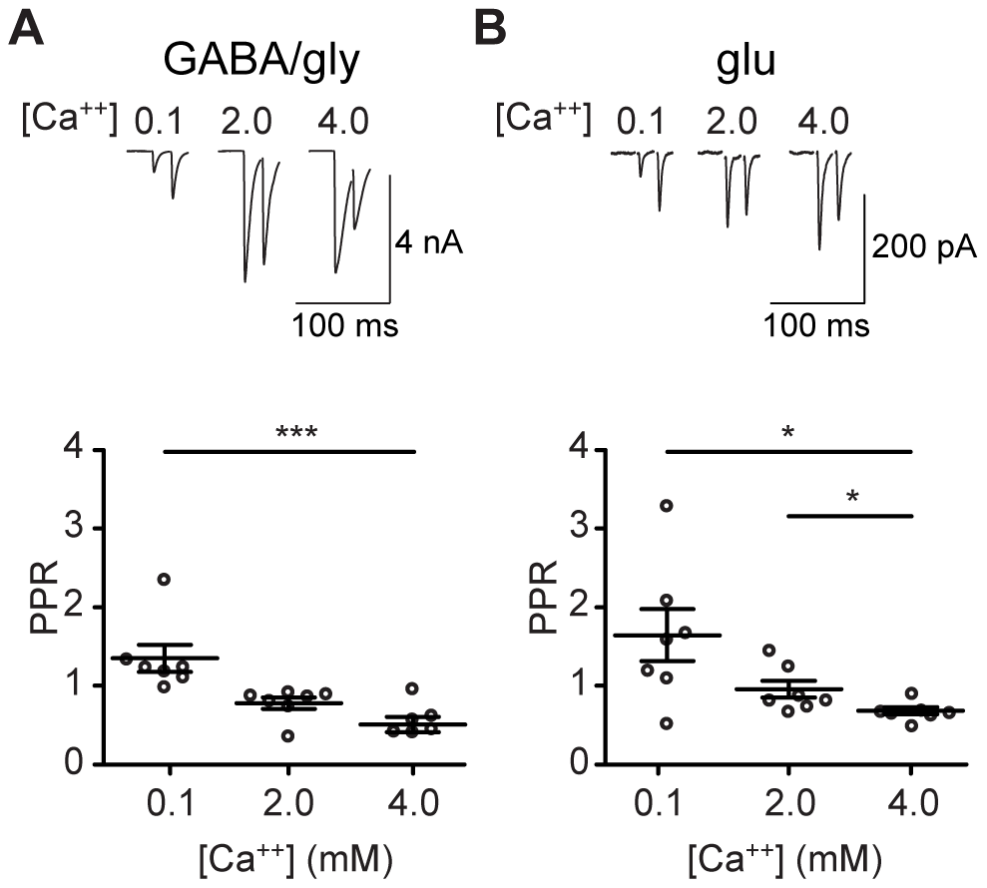
Short-term plasticity is Ca^++^-dependent for both GABA/gly and glutamate. **A**) PPRs for GABA/gly neurotransmission shift from facilitating at low external Ca^++^ (0.1 mM) to depressing at high external Ca^++^ (4.0 mM) (P = 0.0003, F = 12.2, Friedman test, Dunn's post hoc P<0.005). **B**) PPRs for glutamate also shift from facilitating to depressing in a Ca^++^-dependent manner (P = 0.02, F = 7.7, Friedman test, Dunn's post hoc P<0.05). Recordings from P3–5 slices.

We first determined whether and how external Ca^++^ concentration regulates release properties at the onset of the refinement period (P3–8), by measuring the paired pulse ratio (PPR) for GABA/gly and glutamate transmission (50 Hz, P3–5) in low (0.1 mM), putatively normal (2 mM), and high (4 mM) external Ca^++^. We saw that Ca^++^ concentration affected neurotransmitter release in both transmitter systems. Paired-pulse ratios for the GABA/gly component differed significantly in low and high Ca^++^ (P = 0.0003, F = 12.2, Friedman test, Dunn's post hoc P<0.005, Figure 1A). Paired-pulse ratios for the glutamate component differed significantly for all three Ca^++^ regimes (P = 0.02, F = 7.7, Friedman test, Dunn's post hoc P<0.05, Figure 1B). The short-term potentiation of glutamate release seen in low Ca^++^ and short-term depression seen in high Ca^++^ are expected for a synapse at which initial release probability is dependent on Ca^++^ [20]. While this finding is unsurprising for GABA/gly, it helps to establish that anomalous glutamate release from immature GABA/glycinergic MNTB axon terminals occurs through Ca^++^-dependent vesicle fusion (rather than, for example, reversal of membrane transporters).

**Figure 2:**
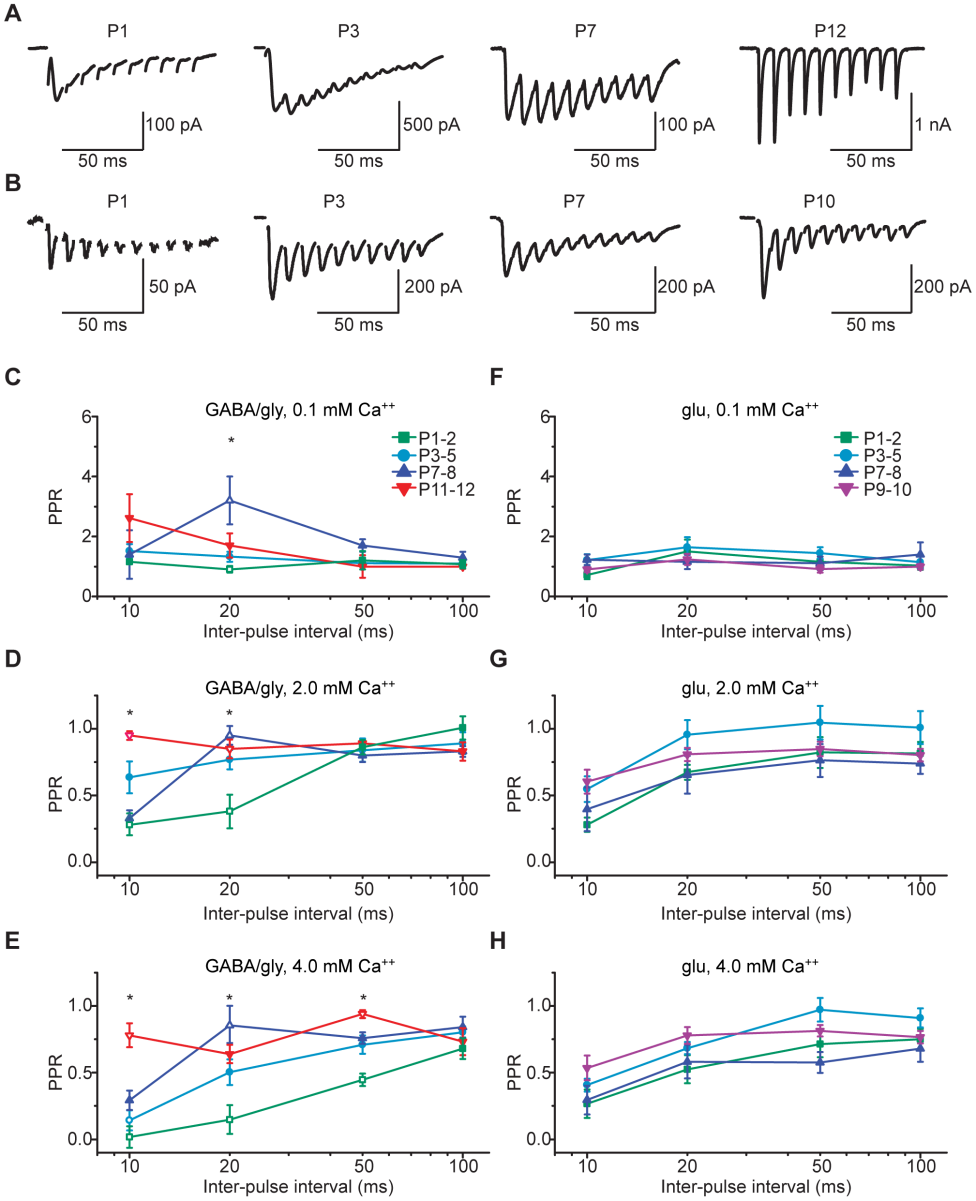
Paired-pulse properties of GABA/gly, but not glutamate, component are significantly altered by developmental age. **A**) Representative GABA/glycine-mediated PSCs (average of 10 traces) recorded in the presence of CNQX (5 µM). **B**) Glutamate-mediated PSCs (average of 10 traces) recorded in the presence of picrotoxin (50 µM), strychnine (10 µM), and cyclothiazide (100 µM). All recordings, in response to 100 Hz electrical stimulation in the MNTB, at a holding potential of −70 mV, with an external Ca^++^ concentration of 2 mM. **C–E**) Summary of PPRs at inter-pulse intervals of 10, 20, 50, and 100 ms for the GABA/gly component in (**C**) 0.1 mM (**D**) 2.0 mM, and (**E**) 4.0 mM external Ca^++^. * P<0.05 (Kruskal-Wallis test; Dunn's post hoc). **F–H**) Summary of PPRs for glutamate component at 10, 20, 50, and 100 ms inter-pulse intervals in (**F**) 0.1 mM, (**G**) 2.0 mM, and (**H**) 4.0 mM external Ca^++^. Open symbols in **C–E** indicate which groups showed significant differences.

We next asked whether release properties for both GABA/gly and glutamate change during development. To address this question, we measured PPRs for inhibitory (GABA/gly) and excitatory (glutamate) neurotransmitters over the entire postnatal pre-hearing period (P1–12) and varied both Ca^++^ concentration and stimulation frequency (10–100 Hz). Note that because GABA and glycine are depolarizing at LSO synapses during much of the pre-hearing period [9], we refer to GABA/gly and glu components rather than IPSCs and EPSCs. At putatively normal (2 mM) Ca^++^, clear developmental trends were seen at 100 Hz stimulation (Fig. 2A–B). Our results for GABA/gly neurotransmission, summarized in Table 1 and depicted in Figure 2A, 2C–E, show that release of GABA/gly is modulated by stimulus frequency as well as by external calcium concentration, and that it undergoes developmental maturation.

**Table 1:** Paired pulse ratios for GABA/gly component.

	Ca^++^ (0.1 mM)				Ca^++^(2.0 mM)				Ca^++^ (4.0 mM)			
	10 Hz	20 Hz	50 Hz	100 Hz	10 Hz	20 Hz	50 Hz	100 Hz	10 Hz	20 Hz	50 Hz	100 Hz
P1–2 (6)	1.1±0.1	1.2±0.3	0.9±0.1*	1.1±0.1	1.0±0.1	0.9±0.1	0.4±0.1*?	0.3±0.1∼	0.7±0.1	0.4±0.1?	0.1±0.1*	0.02±0.1?
P3–5 (7)	1.1±0.1	1.1±0.1	1.3±0.2	1.5±0.2	0.9±0.1	0.8±0.1	0.8±0.1	0.6±0.2	0.8±0.1	0.7±0.1	0.5±0.1	0.1±0.1*
P7–8 (6)	1.3±0.2	1.7±0.2	3.2±0.1*	1.4±0.1	0.8±0.1	0.8±0.1	1.0±0.1*	0.3±0.1	0.8±0.1	0.8±0.1	0.9±0.1*	0.3±0.1
P11–12 (5)	1.0±0.1	1.0±0.4	1.7±0.4	2.6±1.0	0.8±0.1	0.9±0.1	0.9±0.1?	1.0±0.1∼	0.7±0.1	0.9±0.1?	0.6±0.1	0.8±0.1?*

Paired-pulse ratios for the GABA/gly component for different external Ca^++^ concentrations, stimulation frequencies, and postnatal age. Non-parametric 1-way ANOVA (Kruskal-Wallis test) across age revealed significant differences between young and older ages in 2 and 4 mM Ca^++^ (Bonferroni's post hoc *<0.05, ?<0.005, ∼0.0005). The N for each age group is shown in parentheses.

To examine glutamate release properties, we used a separate set of slices from a younger age range (P1–10), as glutamatergic transmission at MNTB-LSO synapses falls off quickly after P8. Unlike the GABA/gly component, PPRs in the glutamate component were similar at all frequencies (Table 2). Additionally, PPRs for glutamatergic transmission were similar at all ages and showed no significant developmental trends (Figures 2B, 2F–H).

**Table 2:** Paired pulse ratios for glutamate component.

	Ca^++^ (0.1 mM)				Ca^++^(2.0 mM)				Ca^++^ (4.0 mM)			
	10 Hz	20 Hz	50 Hz	100 Hz	10 Hz	20 Hz	50 Hz	100 Hz	10 Hz	20 Hz	50 Hz	100 Hz
P1–2 (6)	1.0±0.1	1.2±0.1	1.5±0.4	0.7±0.1	0.8±0.1	0.8±0.1	0.7±0.1	0.3±0.1	0.8±0.1	0.7±0.1	0.5±0.1	0.3±0.1
P3–5 (7)	1.1±0.2	1.4±0.2	1.6±0.3	1.2±0.2	1.0±0.1	1.0±0.1	0.1±0.1	0.5±0.1	0.9±0.1	1.0±0.1	0.7±0.1	0.4±0.1
P7–8 (5)	1.4±0.4	1.1±0.2	1.2±0.2	1.2±0.2	0.7±0.1	0.8±0.1	0.7±0.1	0.4±0.2	0.7±0.1	0.6±0.1	0.6±0.1	0.3±0.1
P9–10 (6)	1.0±0.1	0.9±0.1	1.2±0.2	0.9±0.1	0.8±0.1	0.8±0.1	0.8±0.1	0.6±0.1	0.8±0.1	0.8±0.04	0.8±0.1	0.5±0.1

Paired pulse ratios for the glutamate component for different external Ca^++^ concentrations, stimulation frequencies, and postnatal age. Non-parametric 1-way ANOVA (Kruskal-Wallis test) across age revealed no significant differences. The N of each age group is in parentheses.

**Figure 3:**
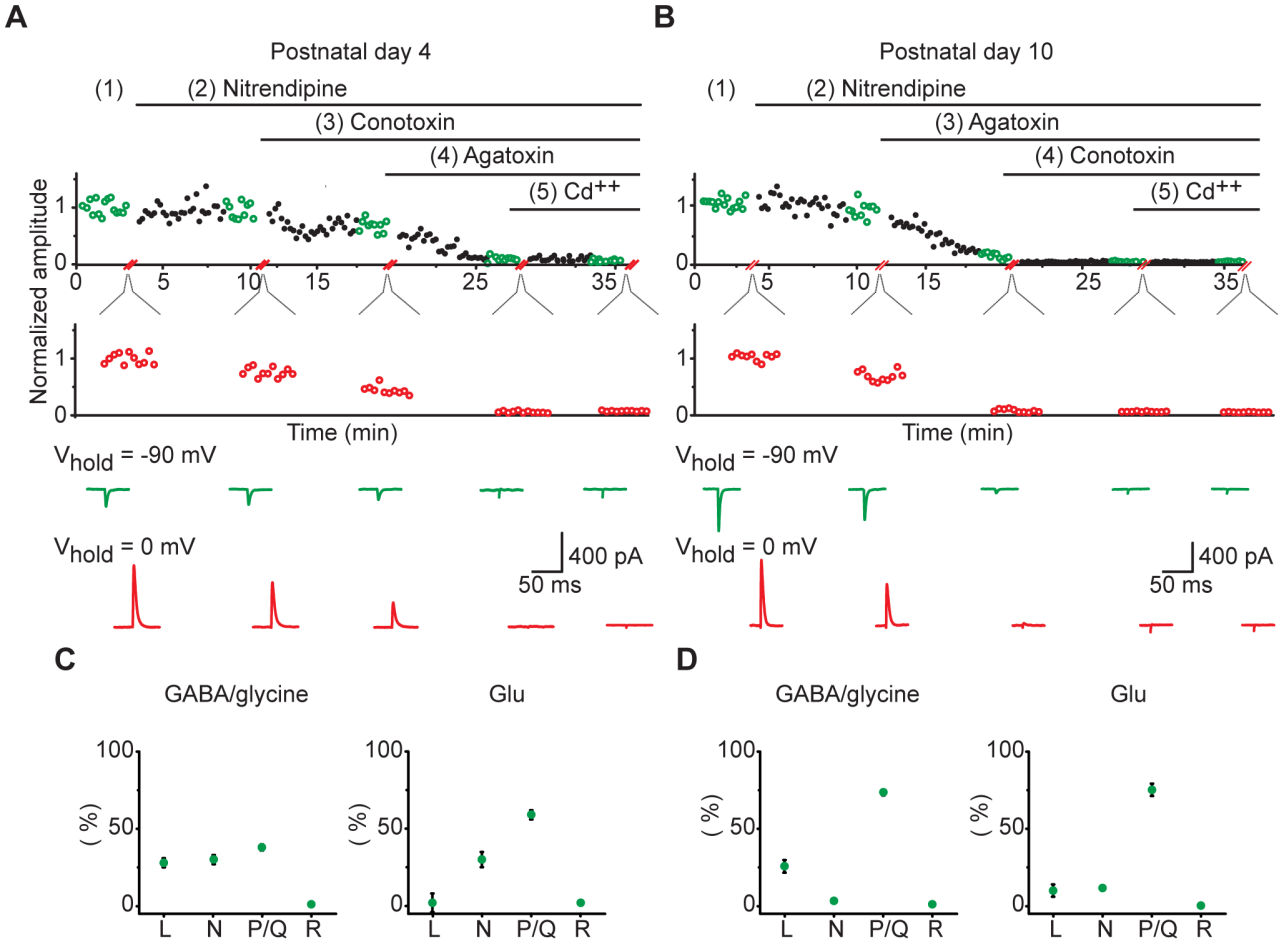
Proportional contribution of VGCC subtypes to GABA/glycine and glutamate transmission. (**A–B**) Representative examples showing how VGCC contribution to GABA/gly and glutamate neurotransmission was determined in (**A**) a P4 slice, and (**B**) a P10 slice. Average GABA/gly (red) and glutamate (green) current traces are shown below, and correspond to the colored points above for each component. (**C–D**) Proportional contribution of each VGCC to GABA/gly and glutamate components for the P4 slice shown in A (**C**), and for the P10 slice shown in B (**D**).

Release properties can be altered in several ways, one of which is through changes in the association of synaptic vesicles with specific voltage-gated Ca^++^ channels (VGCCs). To determine whether different subsets of VGCCs might contribute differentially to neurotransmitter release during different phases of development, in a separate set of slices we recorded responses in the MNTB-LSO pathway in the presence of selective blockers for the L-, N-, and P/Q-type VGCCs. To measure the effect of each toxin on GABA/gly and glutamate components in the same neuron, we set the holding potential alternately to the reversal potential for each component to isolate responses for each component (Figure 3A, 3B). After obtaining baseline responses, we applied the three selective blockers sequentially, in random order, and finally Cd^++^ to test for any residual contribution by R-type VGCCs. In three age groups (P3–5, 7–8 and 9–10), we compared the effects of blockers on both GABA/gly and glutamate, and in an additional set of older slices (P12–15), we examined the effects of VGCC blockers on GABA/gly transmission alone. At no age did we see evidence for a contribution from R-type channels (Figure 3C, 3D) to either GABA/gly or glutamate release. However, L-, N-, and P/Q-types all contributed to transmitter release.

**Figure 4:**
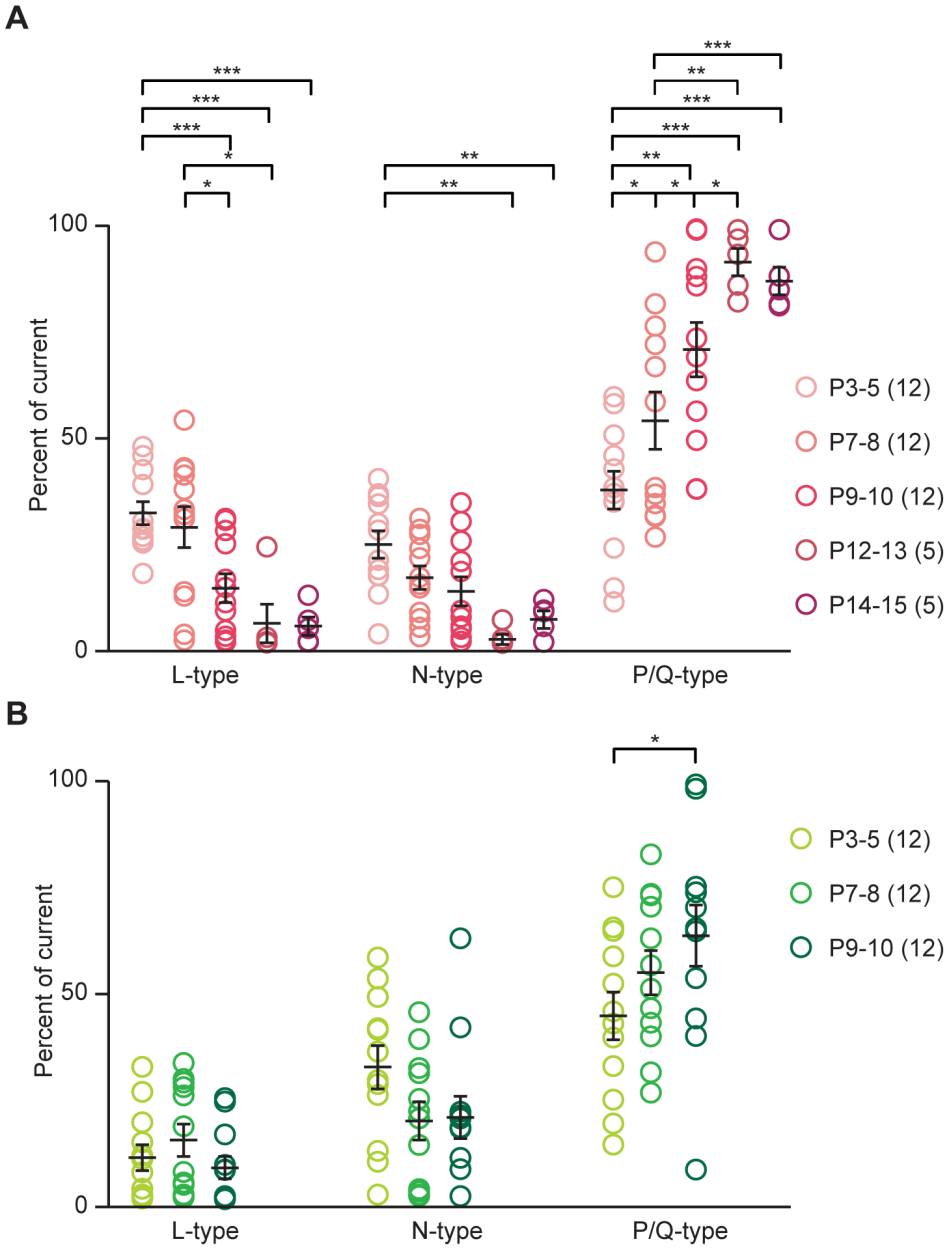
Proportional contribution of VGCC subtypes to neurotransmission between P3 and P15. **A**) Percent contribution of L, N and P/Q-type VGCCs to GABA/gly transmission P3–15 (N for each age group in parentheses). The three VGCCs made significantly different contributions at different ages ([2,123] = 14.76, P<0.0001, 2-way ANOVA; Bonferroni post-tests, * P<0.05, ** P<0.005, *** P<0.0005). **B**) Percent contribution of L, N and P/Q-type VGCCs to glutamate transmission P3–10. L and N-types made similar contributions, while P/Q-type made significantly larger contributions at later ages ([2, 99] = 60.77, P<0.001, 2-way ANOVA; Bonferroni post-tests P<0.05). For ages P3–10, both a GABA/gly and a glutamate component were recorded from each neuron.

For the GABA/gly component, the proportional contributions of L-, N-, and P/Q-subtypes varied with age (Figure 4A). At P3, all three VGCC subtypes contributed similarly (Table 3). From P5 onward, L- and N-type channels contributed progressively less, and P/Q-type channels contributed progressively more, until by hearing onset at P12 GABA/gly release was almost completely under the control of P/Q-type channels ([2,123] = 14.76, p<0.0001, 2-way ANOVA; Table 3). In an additional 4 cells each from younger (P3–5) and older (P14–15) tissue, we blocked glutamatergic transmission pharmacologically (using 1 mM kynurenic acid), and confirmed that these developmental changes in subunit contribution were evident also at normal resting potential (P3–5: L 38±7%; N 25±6%; P/Q 36±8% and P14–15: L 4±1%; N 2±1%; P/Q 91±3%).

**Table 3:** Percent contribution of different VGCC subtypes to GABA/gly and glutamate components.

	GABA/gly			Glutamate		
AGE	L	N	P/Q	L	N	P/Q
P3–5(12)	32±3	25±3	38±5	12±3	35±5	45±6
P7–8(12)	30±5	17±3	55±7	16±4	21±5	55±5
P9–10(12)	15±3	14±3	70±6	10±3	21±5	64±7
P12–13(5)	7±5	3±2	92±5	-	-	-
P14–15(5)	5±2	6±2	86±3	-	-	-

Because glutamatergic transmission in the MNTB-LSO pathway declines rapidly after P8, subtype-specific VGCC contributions to glutamate release were tested until only P10. For the glutamate component, L-type channels made a small and variable contribution at all ages. At P3, the major mediators of release were N- and P/Q-type channels (Table 3; Figure 4B). By P9–10, only about 25% of LSO cells still exhibit glutamatergic responses to MNTB stimulation [35]; for these cells, the contribution to glutamatergic transmission from N-type channels had decreased while that from P/Q-type channels had increased ([2, 99] = 60.77, p<0.001, 2-way ANOVA).

**Figure 5:**
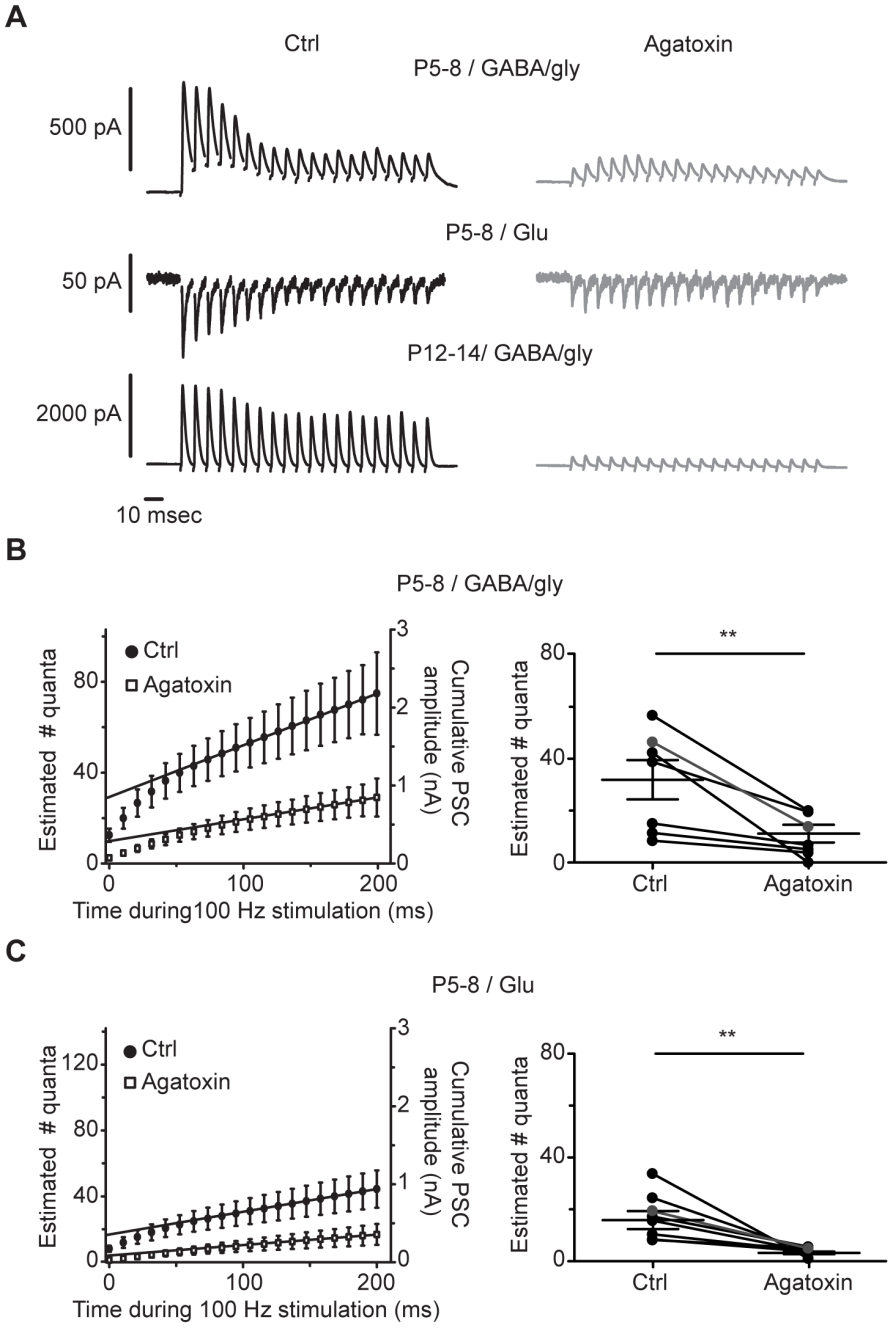
P/Q-type VGCCs influence quantal release for GABA/gly and glutamate component. **A**) PSC traces in control and with ω-agatoxin IVA. GABA/gly and glutamate traces were recorded from the same neuron (P6). GABA/gly traces below from P13 tissue. Each trace shown is the average of 10 responses. **B–C**) Estimated quantal number for GABA/gly component (**B**) and glutamate component (**C**) before and after application of ω-agatoxin IVA at P5–8, calculated from the pooled data on the left and from individual cells on the right. The last 8 data points from the curve were fitted by linear regression and extrapolated to time zero to estimate the readily releasable pool. The cumulative amplitude found by the linear regression was divided by the average amplitude of the sPSC (for GABA/gly 29.0±6.1 pA in control and 26.8±4.7 pA after ω-agatoxin IVA; for glutamate 21.4±2.6 pA in control and 20.0±2.9 pA after ω-agatoxin IVA. Cell in A shown in gray.

To determine the effect of P/Q-type VGCCs on quantal number, we estimated the number of vesicles released after delivery of 20-pulse trains at 100 Hz (Figure 5A). At younger ages (P5–8), blocking P/Q-type channels caused a reduction in the estimate of quantal number for both GABA/gly (Figure 5B: control 30.2±7.4; ω-agatoxin 9.4±3.2; P = 0.0039, W = 36, Wilcoxon test), and glutamate transmission (Figure 5C: control 16.3±3.5; ω-agatoxin 4.3±0.5; P = 0.0078, W = 28, Wilcoxon test). At older ages (P12–15) this analysis was not possible for either the GABA/gly component, which was nearly completely abolished by ω-agatoxin IVA (Figure 5A, lower right), or for the glutamate component, which is nearly absent at this age.

**Figure 6:**
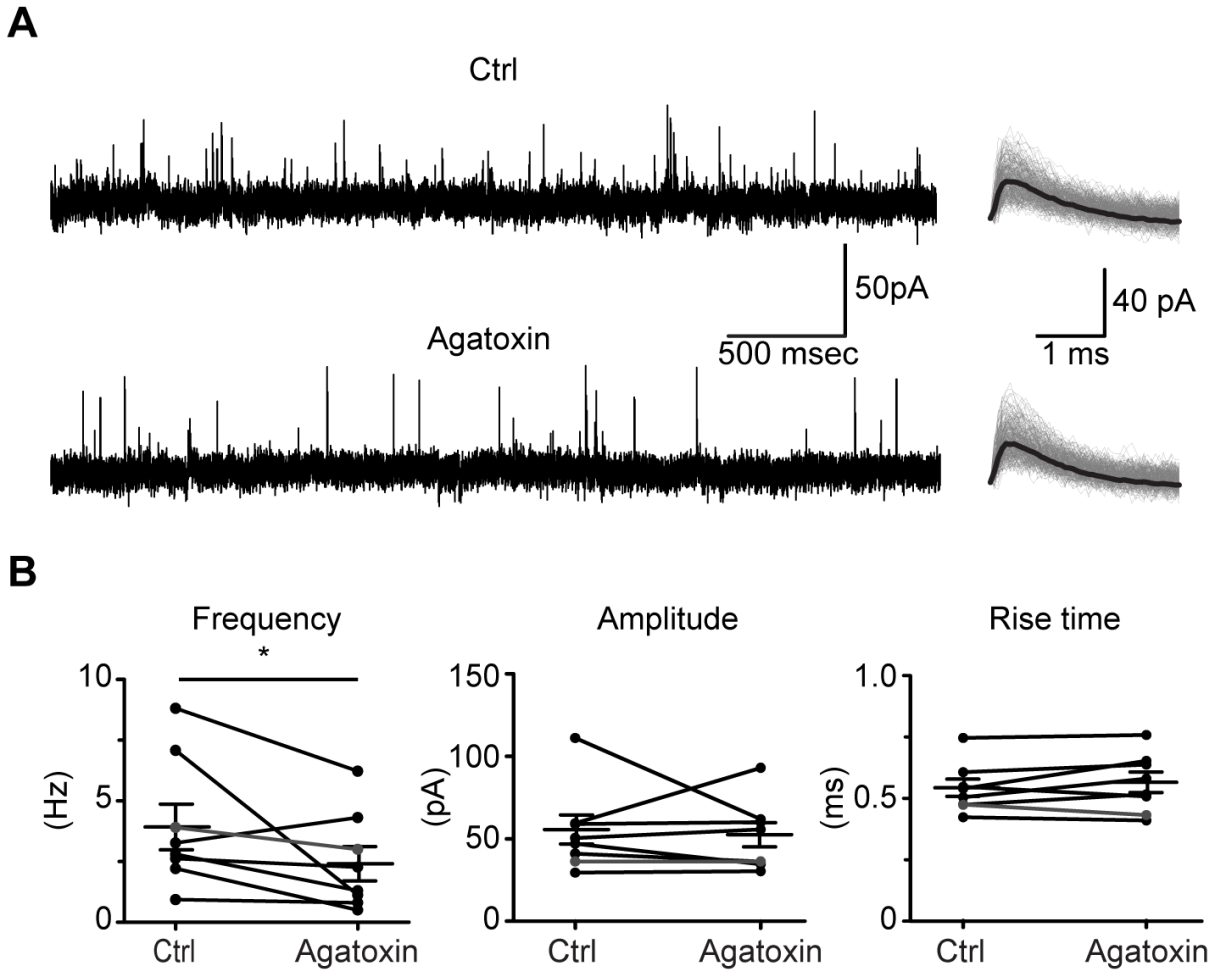
P/Q-type Ca^++^ channels mediate evoked miniature events at older ages. **A**) (Left) Example current trace collected after 100 Hz stimulation (P12 cell), showing reduction in sPSC frequency after application of the P/Q-type antagonist ω-agatoxin IVA. (Right) Superimposed sPSCs (150 events) in control and in ω-agatoxin IVA, for the neuron shown in left; average traces in black. **B**) Mean frequency, but not amplitude or rise time, of sPSCs was affected by P/Q-block with ω-agatoxin IVA. Black lines connect control and drug measurements from individual cells. Cell in A shown in gray.

As our measure for presynaptic effects is a postsynaptic response, we wished to confirm that the P/Q antagonist did not have measurable postsynaptic effects. Therefore, in a separate set of slices (P12–15; N = 8) we recorded “asynchronous spontaneous PSCs” (aPSCs) induced after 100 Hz stimulation, both with and without ω-agatoxin IVA application (Figure 6A), under the rationale that if the effect of ω-agatoxin IVA was purely presynaptic we would see a reduction in aPSC frequency but no changes in amplitude or rise time. Note that by shortly after hearing onset the aPSC was entirely glycinergic, as strychnine administration completely blocked aPSCs (N = 8, P12–15, data not shown). The frequency of aPSCs declined after block of P/Q-type channels (mean frequency before and after ω-agatoxin: 3.9±0.9 Hz and 2.4±0.7 Hz; P = 0.0273, W = 28, Wilcoxon test, Figure 6B; median ISIs before and after ω-agatoxin: 253.4 ms and 405.2 ms). Amplitude of the aPSC, however, was not affected by P/Q block (before and after ω-agatoxin: 54.2±8.9 pA and 51.0±7.4 pA; P = 0.9453, W = 2, Wilcoxon test, Figure 6B), and rise time was also unaffected by P/Q block (before and after ω-agatoxin: 0.54±0.03 ms and 0.56±0.04 ms; P = 0.3828, W = −14, Wilcoxon test, Figure 6B). The decrease in frequency without concomitant effects on amplitude or kinetics confirmed that ω-agatoxin application did not modulate post-synaptic responses, and therefore that the effect was primarily on presynaptic release.

**Figure 7:**
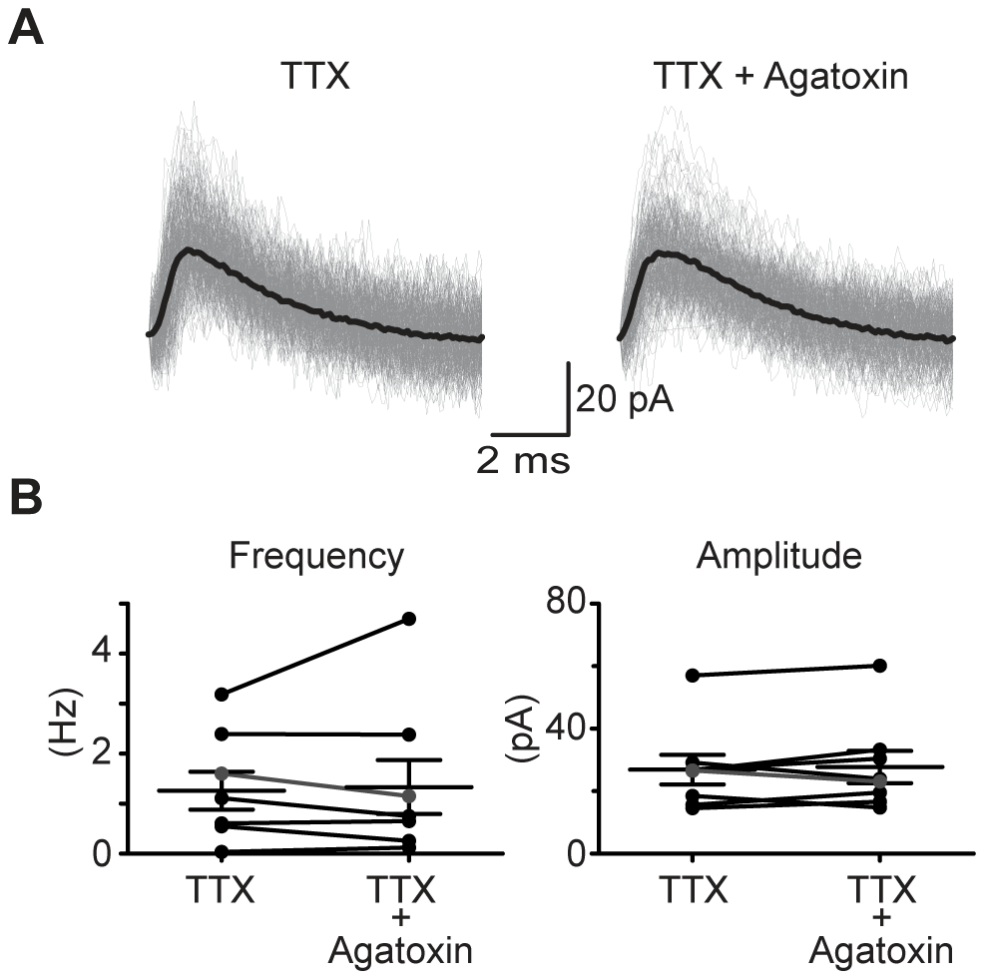
Spontaneous miniature events occur independent of P/Q activation. **A**) Example miniature PSCs recorded in TTX, before and after ω-agatoxin IVA, in a P12 slice. **B**) Mean mPSC frequency (left) and amplitude (right) for each neuron recorded before and after P/Q block. N = 8 cells, P12–15. Cell in A shown in gray.

After application of ω-agatoxin IVA, aPSC frequency was reduced but not abolished. To determine whether P/Q-type VGCCs might contribute to normal spontaneous release, in a separate set of slices P12–15 (N = 8) we recorded miniature spontaneous events (mPSCs) in TTX (1 µM) both before and after ω-agatoxin administration (Figure 7A). Unlike our findings with asynchronous spontaneous release, P/Q block caused no change in either mPSC frequency (1.2±0.3 Hz before and 1.3±0.5 after ω-agatoxin; P = 0.94, W = 2, Wilcoxon test, Figure 7B) or mPSC amplitude (before and after ω-agatoxin: 26.8±5 pA and 27.7±5 pA; P = 0.84, W = −4, Wilcoxon test, Figure 7B). These results support a model in which P/Q-type VGCCs control evoked or synchronous release, and spontaneous release is independent of P/Q VGCCs.

## Discussion

In the immature MNTB-LSO pathway, release of both GABA/gly and glutamate depends on Ca^++^. While this result may be obvious for glycinergic transmission, and was expected for glutamate transmission, this is nevertheless the first confirmation that the anomalous glutamate release that occurs transiently in this immature inhibitory pathway is subject to normal rules for neurotransmission at chemical synapses. For both GABA/gly and glutamate, the increase in paired-pulse depression with increasing Ca^++^, generally seen as a reflection of increasing initial release probability, supports the idea that invasion of the nerve terminal by an action potential results in release of only a fraction of the readily releasable pool.

We examined the influence of external [Ca^++^] and stimulus frequency on synaptic transmission during development of the MNTB-LSO pathway. The GABA/gly component showed a strong maturational trend: though this component depressed strongly around birth, by well before hearing onset it was able to follow at frequencies above 100 Hz. In agreement with previous room temperature recordings in the mouse and rat MNTB-LSO pathway [35], [36], for stimulus frequencies of 50–100 Hz we found significantly greater paired-pulse depression at the earliest (P1–2) than the oldest (P11–12) ages. However, we were also able to see a rapid decline in paired-pulse depression with developmental age. In comparison with GABA/glycine release, glutamate release was less sensitive at all ages to stimulus frequency and showed less robust maturational changes.

Paired-pulse ratios can be affected by several factors, but are especially strongly influenced by presynaptic calcium, and hence by activation and inactivation of presynaptic VGCCs [37], [38]. We found a clear developmental trend in the relative contribution of different VGCCs to GABA/gly neurotransmission over the first two postnatal weeks. As co-administration of nitrendipine, ω-conotoxin and ω-agatoxin abolished the PSC in all cases, we conclude that R- and T-type VGCCs do not mediate transmitter release in the immature MNTB-LSO pathway. Channels of type L, N, and P/Q, however, do mediate release, and their relative contributions change during the first two postnatal weeks. Whereas L- and N- type made small contributions to transmitter release neonatally, by hearing onset at P12 the dominant channel type is P/Q.

These results differ slightly from results in the immature MNTB, where N- and R-type channels contribute and P/Q channels dominate at the calyx of Held [27]. They also differ from results in the MNTB-LSO pathway of mice from a 129 background, in which L-, N- and P/Q-types all contribute to GABA/gly release around hearing onset, and P/Q dominates only slightly [29]. These differences could reflect species/strain differences, or perhaps differential temperature-dependent effects on VGCC subtypes, as temperature can profoundly influence VGCC function [39]–[42]. The number of conditions in this study precludes direct comparison of N-type contribution to GABA/gly versus glutamate release. Nevertheless, it is interesting to note the suggestion in these results (Fig. 4) that at young ages N-type channels may play a greater role in glutamate than in GABA/gly release. Similarly, in the neighboring MSO nucleus N-type VGCCs appear to mediate a larger proportion of glutamate than of GABA/gly inputs [28]. A noticeable difference between results in the rat LSO and MSO is that after hearing onset glycinergic transmission is more dependent on N-type VGCCs in the MSO than in the LSO (∼20% vs ∼0%). While some discrepancies might be explained by temperature differences, an alternative possibility is that the expression of different VGCCs reflects maturation to optimize the qualitatively different kinds of auditory processing performed by these two nuclei.

Overall, glutamate release showed less robust maturational changes than GABA/gly. While the larger variance and weaker trend toward P/Q dominance seen for glutamate release could result in part from the smaller absolute amplitudes of glutamate responses, they may also reflect a distinct, developmental function for glutamate. Glutamatergic transmission is understood to be required for developmental synapse elimination in the MNTB-LSO pathway, as VGLUT3 knockout mice exhibit impaired refinement of this circuit [43]. If the function of glutamate release in the immature MNTB-LSO pathway is less to relay auditory information and more to mediate refinement during a finite developmental period, then specific maturation of glutamate release might be both unnecessary and absent.

We estimated the number of quanta released for GABA/gly and for glutamate in control and after the P/Q-type blocker using a method that takes into account the pool of readily releasable vesicles after a short train of high frequency stimulation [34], [44]. At P5–8, ω-agatoxin IVA decreased quantal number by 69% and 73% for GABA/gly and glutamate components. At P12–15, reduced GABA/gly transmission after high frequency trains so much that that quantal number could not be estimated. Therefore, at older ages (P12–15) we analyzed spontaneous events recorded before and after administration of ω-agatoxin IVA. The P/Q blocker reduced sPSC frequency but not amplitude or rise time, consistent with an effect on release probability.

In summary, whereas synapses in the phase-processing pathway have been shown to use additional mechanisms [12], [45] to ensure rapid synaptic communication, our results support the idea that synaptic terminals in the intensity-processing pathway between the MNTB and LSO undergo similar developmental maturation of calcium-dependent release. Thus, synapses throughout the auditory brainstem mature to favor P/Q-mediated VGCC-dependent release in response to spiking activity in the axon terminal.

## References

[1] Boudreau JC, Tsuchitani C (1968) Binaural interaction in the cat superior olive S segment. J Neurophysiol 31: 442–454. 5687764 10.1152/jn.1968.31.3.442

[2] Caird D, Klinke R (1983) Processing of binaural stimuli by cat superior olivary complex neurons. Exp Brain Res 52: 385–399. 6653700 10.1007/BF00238032

[3] Cant NB, Casseday JH (1986) Projections from the anteroventral cochlear nucleus to the lateral and medial superior olivary nuclei. J Comp Neurol 247: 457–476. 3722446 10.1002/cne.902470406

[4] Wu SH, Kelly JB (1992) Synaptic pharmacology of the superior olivary complex studied in mouse brain slice. J Neurosci 12: 3084–3097. 1494947 10.1523/JNEUROSCI.12-08-03084.1992 PMC6575641

[5] Kim G, Kandler K (2003) Elimination and strengthening of glycinergic/GABAergic connections during tonotopic map formation. Nat Neurosci 6: 282–290. 12577063 10.1038/nn1015

[6] Kotak VC, Korada S, Schwartz IR, Sanes DH (1998) A developmental shift from GABAergic to glycinergic transmission in the central auditory system. J Neurosci 18: 4646–4655. 9614239 10.1523/JNEUROSCI.18-12-04646.1998 PMC6792682

[7] Nabekura J, Katsurabayashi S, Kakazu Y, Shibata S, Matsubara A, et al (2004) Developmental switch from GABA to glycine release in single central synaptic terminals. Nat Neurosci 7: 17–23. 14699415 10.1038/nn1170

[8] Kandler K, Friauf E (1995) Development of glycinergic and glutamatergic synaptic transmission in the auditory brainstem of perinatal rats. J Neurosci 15: 6890–6904. 7472446 10.1523/JNEUROSCI.15-10-06890.1995 PMC6578015

[9] Ehrlich I, Lohrke S, Friauf E (1999) Shift from depolarizing to hyperpolarizing glycine action in rat auditory neurones is due to age-dependent Cl- regulation. J Physiol 520 Pt 1: 121–137. 10517806 10.1111/j.1469-7793.1999.00121.x PMC2269580

[10] Gillespie DC, Kim G, Kandler K (2005) Inhibitory synapses in the developing auditory system are glutamatergic. Nat Neurosci 8: 332–338. 15746915 10.1038/nn1397

[11] Cooper AP, Gillespie DC (2011) Synaptotagmins I and II in the developing rat auditory brainstem: Synaptotagmin I is transiently expressed in glutamate-releasing immature inhibitory terminals. J Comp Neurol 519: 2417–2433. 21456023 10.1002/cne.22634

[12] Fedchyshyn MJ, Wang LY (2005) Developmental transformation of the release modality at the calyx of Held synapse. J Neurosci 25: 4131–4140. 15843616 10.1523/JNEUROSCI.0350-05.2005 PMC6724960

[13] Mochida S, Few AP, Scheuer T, Catterall WA (2008) Regulation of presynaptic Ca(V)2.1 channels by Ca2+ sensor proteins mediates short-term synaptic plasticity. Neuron 57: 210–216. 18215619 10.1016/j.neuron.2007.11.036

[14] Fuhrmann G, Segev I, Markram H, Tsodyks M (2002) Coding of temporal information by activity-dependent synapses. J Neurophysiol 87: 140–148. 11784736 10.1152/jn.00258.2001

[15] Abbott LF, Regehr WG (2004) Synaptic computation. Nature 431: 796–803. 15483601 10.1038/nature03010

[16] Kandler K, Clause A, Noh J (2009) Tonotopic reorganization of developing auditory brainstem circuits. Nat Neurosci 12: 711–717. 19471270 10.1038/nn.2332 PMC2780022

[17] Llinas R, Yarom Y (1981) Electrophysiology of mammalian inferior olivary neurones in vitro. Different types of voltage-dependent ionic conductances. J Physiol 315: 549–567. 6273544 10.1113/jphysiol.1981.sp013763 PMC1249398

[18] Mintz IM, Adams ME, Bean BP (1992) P-type calcium channels in rat central and peripheral neurons. Neuron 9: 85–95. 1321648 10.1016/0896-6273(92)90223-z

[19] Olivera BM, Miljanich GP, Ramachandran J, Adams ME (1994) Calcium channel diversity and neurotransmitter release: the omega-conotoxins and omega-agatoxins. Annu Rev Biochem 63: 823–867. 7979255 10.1146/annurev.bi.63.070194.004135

[20] Randall A, Tsien RW (1995) Pharmacological dissection of multiple types of Ca2+ channel currents in rat cerebellar granule neurons. J Neurosci 15: 2995–3012. 7722641 10.1523/JNEUROSCI.15-04-02995.1995 PMC6577783

[21] Tsien RW, Lipscombe D, Madison DV, Bley KR, Fox AP (1988) Multiple types of neuronal calcium channels and their selective modulation. Trends Neurosci 11: 431–438. 2469160 10.1016/0166-2236(88)90194-4

[22] Perez-Reyes E, Cribbs LL, Daud A, Lacerda AE, Barclay J, et al (1998) Molecular characterization of a neuronal low-voltage-activated T-type calcium channel. Nature 391: 896–900. 9495342 10.1038/36110

[23] Mintz IM, Sabatini BL, Regehr WG (1995) Calcium control of transmitter release at a cerebellar synapse. Neuron 15: 675–688. 7546746 10.1016/0896-6273(95)90155-8

[24] Inchauspe CG, Martini FJ, Forsythe ID, Uchitel OD (2004) Functional compensation of P/Q by N-type channels blocks short-term plasticity at the calyx of held presynaptic terminal. J Neurosci 24: 10379–10383. 15548652 10.1523/JNEUROSCI.2104-04.2004 PMC6730293

[25] Hefft S, Jonas P (2005) Asynchronous GABA release generates long-lasting inhibition at a hippocampal interneuron-principal neuron synapse. Nat Neurosci 8: 1319–1328. 16158066 10.1038/nn1542

[26] Salgado H, Tecuapetla F, Perez-Rosello T, Perez-Burgos A, Perez-Garci E, et al (2005) A reconfiguration of CaV2 Ca2+ channel current and its dopaminergic D2 modulation in developing neostriatal neurons. J Neurophysiol 94: 3771–3787. 16120665 10.1152/jn.00455.2005

[27] Iwasaki S, Takahashi T (1998) Developmental changes in calcium channel types mediating synaptic transmission in rat auditory brainstem. J Physiol 509 (Pt 2): 419–423. 9575291 10.1111/j.1469-7793.1998.419bn.x PMC2230976

[28] Barnes-Davies M, Owens S, Forsythe ID (2001) Calcium channels triggering transmitter release in the rat medial superior olive. Hear Res 162: 134–145. 11707360 10.1016/s0378-5955(01)00378-1

[29] Giugovaz-Tropper B, Gonzalez-Inchauspe C, Di Guilmi MN, Urbano FJ, Forsythe ID, et al (2011) P/Q-type calcium channel ablation in a mice glycinergic synapse mediated by multiple types of Ca(2)+ channels alters transmitter release and short term plasticity. Neuroscience 192: 219–230. 21718757 10.1016/j.neuroscience.2011.06.021

[30] Ene FA, Kullmann PH, Gillespie DC, Kandler K (2003) Glutamatergic calcium responses in the developing lateral superior olive: receptor types and their specific activation by synaptic activity patterns. J Neurophysiol 90: 2581–2591. 12853437 10.1152/jn.00238.2003

[31] Hirtz JJ, Boesen M, Braun N, Deitmer JW, Kramer F, et al (2011) Cav1.3 calcium channels are required for normal development of the auditory brainstem. J Neurosci 31: 8280–8294. 21632949 10.1523/JNEUROSCI.5098-10.2011 PMC6622878

[32] Woodin MA, Ganguly K, Poo MM (2003) Coincident pre- and postsynaptic activity modifies GABAergic synapses by postsynaptic changes in Cl- transporter activity. Neuron 39: 807–820. 12948447 10.1016/s0896-6273(03)00507-5

[33] Stocker JW, Nadasdi L, Aldrich RW, Tsien RW (1997) Preferential interaction of omega-conotoxins with inactivated N-type Ca2+ channels. J Neurosci 17: 3002–3013. 9096136 10.1523/JNEUROSCI.17-09-03002.1997 PMC6573648

[34] Schneggenburger R, Meyer AC, Neher E (1999) Released fraction and total size of a pool of immediately available transmitter quanta at a calyx synapse. Neuron 23: 399–409. 10399944 10.1016/s0896-6273(00)80789-8

[35] Case DT, Gillespie DC (2011) Pre- and postsynaptic properties of glutamatergic transmission in the immature inhibitory MNTB-LSO pathway. J Neurophysiol 106: 2570–2579. 21832038 10.1152/jn.00644.2010

[36] Kim G, Kandler K (2010) Synaptic changes underlying the strengthening of GABA/glycinergic connections in the developing lateral superior olive. Neuroscience 171: 924–933. 20888399 10.1016/j.neuroscience.2010.09.054 PMC2987552

[37] Forsythe ID, Tsujimoto T, Barnes-Davies M, Cuttle MF, Takahashi T (1998) Inactivation of presynaptic calcium current contributes to synaptic depression at a fast central synapse. Neuron 20: 797–807. 9581770 10.1016/s0896-6273(00)81017-x

[38] Xu J, Wu LG (2005) The decrease in the presynaptic calcium current is a major cause of short-term depression at a calyx-type synapse. Neuron 46: 633–645. 15944131 10.1016/j.neuron.2005.03.024

[39] Peloquin JB, Doering CJ, Rehak R, McRory JE (2008) Temperature dependence of Cav1.4 calcium channel gating. Neuroscience 151: 1066–1083. 18206315 10.1016/j.neuroscience.2007.11.053

[40] Iftinca M, McKay BE, Snutch TP, McRory JE, Turner RW, et al (2006) Temperature dependence of T-type calcium channel gating. Neuroscience 142: 1031–1042. 16935432 10.1016/j.neuroscience.2006.07.010

[41] McAllister-Williams RH, Kelly JS (1995) The temperature dependence of high-threshold calcium channel currents recorded from adult rat dorsal raphe neurones. Neuropharmacology 34: 1479–1490. 8606795 10.1016/0028-3908(95)00130-x

[42] Rosen AD (1996) Temperature modulation of calcium channel function in GH3 cells. Am J Physiol 271: C863–868. 8843716 10.1152/ajpcell.1996.271.3.C863

[43] Noh J, Seal RP, Garver JA, Edwards RH, Kandler K (2010) Glutamate co-release at GABA/glycinergic synapses is crucial for the refinement of an inhibitory map. Nat Neurosci 13: 232–238. 20081852 10.1038/nn.2478 PMC2832847

[44] Schneggenburger R, Sakaba T, Neher E (2002) Vesicle pools and short-term synaptic depression: lessons from a large synapse. Trends Neurosci 25: 206–212. 11998689 10.1016/s0166-2236(02)02139-2

[45] Wang LY, Fedchyshyn MJ, Yang YM (2009) Action potential evoked transmitter release in central synapses: insights from the developing calyx of Held. Mol Brain 2: 36. 19939269 10.1186/1756-6606-2-36 PMC2794261

